# How to activate the glutes best? Peak muscle activity of acceleration-specific pre-activation and traditional strength training exercises

**DOI:** 10.1007/s00421-023-05400-3

**Published:** 2024-01-27

**Authors:** Maximilian Goller, Oliver J. Quittmann, Tobias Alt

**Affiliations:** 1 Institute of Movement and Neurosciences, German Sport University Cologne, Am Sportpark Müngersdorf 6, 50933 Cologne, Germany; 2 Department of Biomechanics, Performance Analysis and Strength and Conditioning, Olympic Training and Testing Centre Westphalia, Dortmund, Germany

**Keywords:** Activation training, Gluteus maximus, Gluteus medius, Unilateral strength training, Thigh separation, Reciprocal inhibition

## Abstract

**Purpose:**

Isometric training and pre-activation are proven to enhance acceleration performance. However, traditional strength training exercises do not mirror the acceleration-specific activation patterns of the gluteal muscles, characterized by ipsilateral hip extension during contralateral hip flexion. Therefore, the aim of the study was to determine gluteal muscle activity of acceleration-specific exercises compared to traditional strength training exercises.

**Methods:**

In a cross-sectional study design, the peak electromyographic activity of two acceleration-specific exercises was investigated and compared to two traditional strength training exercises each for the gluteus maximus and medius. Twenty-four participants from various athletic backgrounds (13 males, 11 females, 26 years, 178 cm, 77 kg) performed four gluteus maximus [half-kneeling glute squeeze (HKGS), resisted knee split (RKS), hip thrust (HT), split squat (SS)] and four gluteus medius [resisted prone hip abduction (RPHA), isometric clam (IC), side-plank with leg abduction (SP), resisted side-stepping (RSS)] exercises in a randomized order.

**Results:**

The RKS (*p* = 0.011, *d* = 0.96) and the HKGS (*p* = 0.064, *d* = 0.68) elicited higher peak gluteus maximus activity than the SS with large and moderate effects, respectively. No significant differences (p > 0.05) were found between the HT, RKS and HKGS. The RPHA elicited significantly higher gluteus medius activity with a large effect compared to RSS (*p* < 0.001, *d* = 1.41) and a moderate effect relative to the SP (*p* = 0.002, *d* = 0.78).

**Conclusion:**

The acceleration-specific exercises effectively activate the gluteal muscles for pre-activation and strength training purposes and might help improve horizontal acceleration due to their direct coordinative transfer.

**Supplementary Information:**

The online version contains supplementary material available at 10.1007/s00421-023-05400-3.

## Introduction

The interest in isometric training has risen over the last few years, both in sports practice and in research (Bartolomei et al. 2017; Cannon et al. 2022; Oranchuk et al. 2019). High-intensity isometric training allows long-term improvements in muscular activation, maximum force production (Oranchuk et al. 2019) and tendon stiffness (Bohm et al. 2015) as well as short-term performance enhancements after pre-activation tasks (Bartolomei et al. 2017; Seitz and Haff 2016; Wilson et al. 2013). Post-activation improvements have been identified in horizontal acceleration performance (Chatzopoulos et al. 2007; Smith et al. 2014) which is a key determinant in a large number of sports (Wild et al. 2022). A high coordinative transfer of the pre-activation task to horizontal acceleration is suggested to increase the benefits of post-activation potentiation (PAP) (Dello Iacono et al. 2018; Smith et al. 2014). However, most of the traditional pre-activation and strength training exercises such as squats, step-ups, hip thrusts, split squats, or Olympic weightlifting variations do not reflect the specific activation patterns that are present during horizontal acceleration. The term ’acceleration-specificity’ implies high muscle activity, high tendon stretch and the synergistic interaction of both legs during a sports-specific joint angle configuration, i.e. ipsilateral hip extension with high contralateral hip flexion (’hip-extensor driven’ movement pattern).

The thigh separation angle is described as a crucial factor for horizontal power generation (Walker et al. 2021). Hip flexor co-contraction causes reciprocal inhibition of the gluteus maximus (GMAX) in the stance leg. This impairs ipsilateral hip extension and contralateral hip flexion which results in a decreased thigh separation (Mills et al. 2015). Higher GMAX activity is suggested to decrease co-contraction of the hip flexor muscles and thus enhance the thigh separation angle (Neumann 2010). A recently published framework introduced the half-kneeling glute squeeze exercise to improve the acceleration-specific GMAX activation (Alt et al. 2022). Although the half-kneeling glute squeeze and the similar resisted knee split exercise are practically approved by elite Olympic athletes from different sports, they have not yet been examined.

A major synergistic muscle to support hip extension is the gluteus medius (GMED) (Neumann 2010), which has been identified as an important generator of horizontal forces during accelerated sprinting (Pandy et al. 2021). Therefore, it is imperative to improve his proper function to enhance acceleration-specific activation patterns. However, most training approaches for the GMED are based on low intensities and high volume instead of high activity levels (Macadam et al. 2015; Moore et al. 2020). Consequently, the resisted prone hip abduction was designed and the isometric clam exercise was modified to enable high GMED intensities. This might help athletes to gradually modulate activity levels and thus enhance their range of motion of active thigh separation.

In addition to performance enhancement, high GMAX and GMED activity is important for effective prevention and rehabilitation of lower extremity injuries (Chumanov et al. 2012; Distefano et al. 2009; Mills et al. 2015). Reciprocal inhibition of the GMAX and weakness of the GMED have been shown to increase the injury risk of the anterior cruciate ligament, the hip flexor, hamstring and adductor muscles, among others (Mills et al. 2015; Presswood et al. 2008). Furthermore, the acceleration-specific exercises could help elicit high GMAX and GMED activity with low joint stress, which might help maintain gluteal strength after lower extremity injuries (Cambridge et al. 2012). Due to the minimal equipment requirements, they can easily be incorporated in rehabilitation and pre-activation settings at any facility with a high number of athletes in a time-efficient manner.

To the best of our knowledge, no study has investigated muscular activity during these exercises. The objective of this investigation is to examine the gluteal activity of the four acceleration-specific exercises and to compare the GMAX exercises to the hip thrust and split squat and the GMED exercises to the side-plank with leg abduction and resisted side-stepping. The hypothesis is as follows:

Peak muscle activity of GMAX and GMED is higher in acceleration-specific exercises compared to traditional strength training exercises.

## Methods

### Participants

Twenty-four participants (25.6 ± 3.5 years, 177.7 ± 9.6 cm, 76.7 ± 13.9 kg, 13 males, 11 females) from various athletic backgrounds (supplementary data A.1) with a mean strength training experience of 5.5 ± 3.0 years were recruited for the investigation. Inclusion criteria required that participants were at least 18 years of age, were not acutely affected by any form of injuries, had at least one year of strength training experience and performed regular training two to three times a week. All participants were informed about the aims of the study, the procedure of electromyography (EMG) and provided written consent to their voluntary participation. The investigation was approved by the local ethics commission of the German Sport University Cologne (No. 168/2021) and met all requirements of the Declaration of Helsinki.

### Study design

**Fig. 1:**
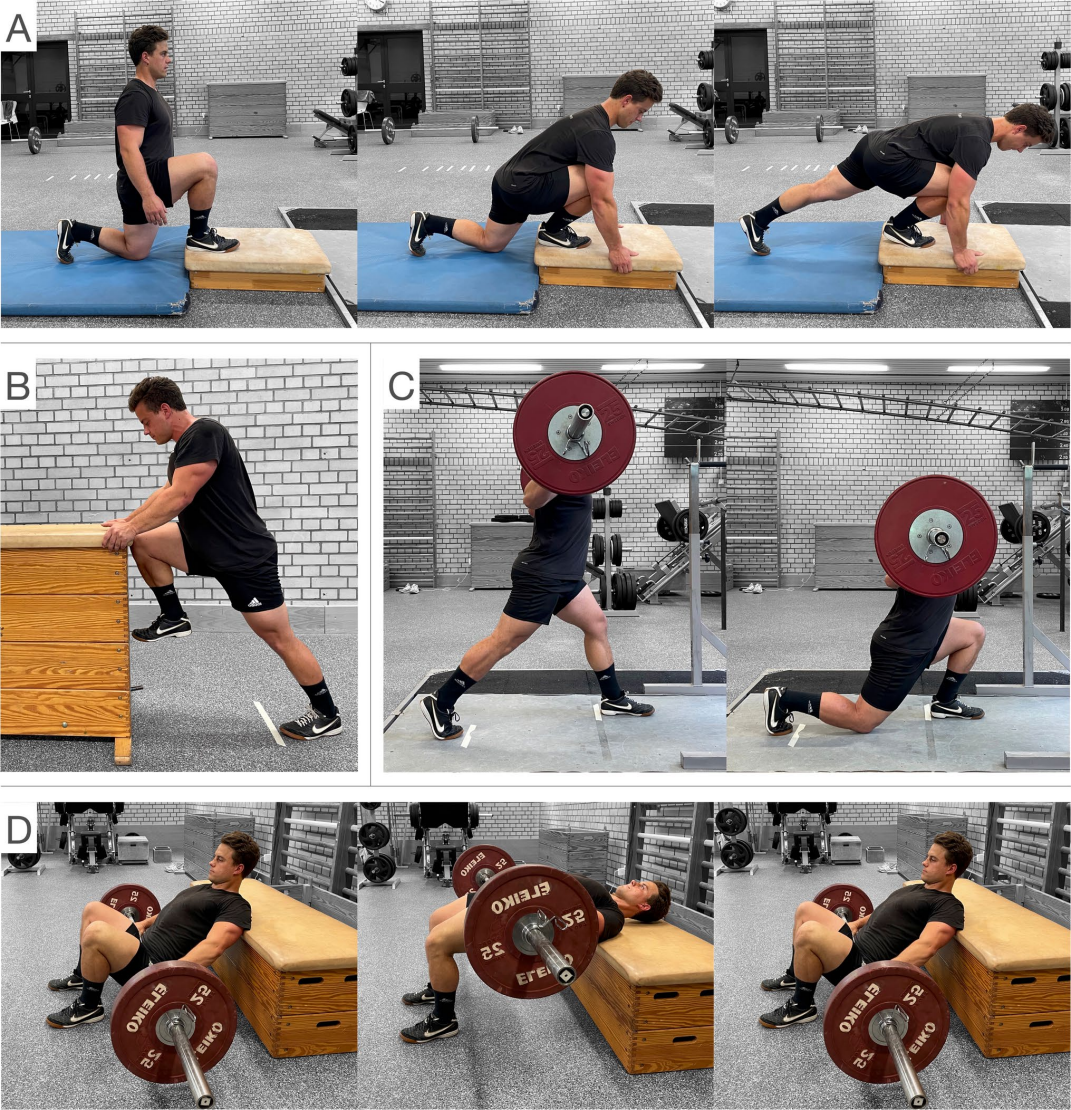
Representative illustration of the four gluteus maximus exercises: **A** Half-kneeling glute squeeze, static phase one (left), increasing hip flexion in phase two (middle) and extension of the dominant leg in phase three (right); **B** resisted knee split; **C** split squat, starting position (left) and position after the eccentric phase (right); **D** hip thrust, starting (left), isometric hold (middle) and end position (right)

**Fig. 2:**
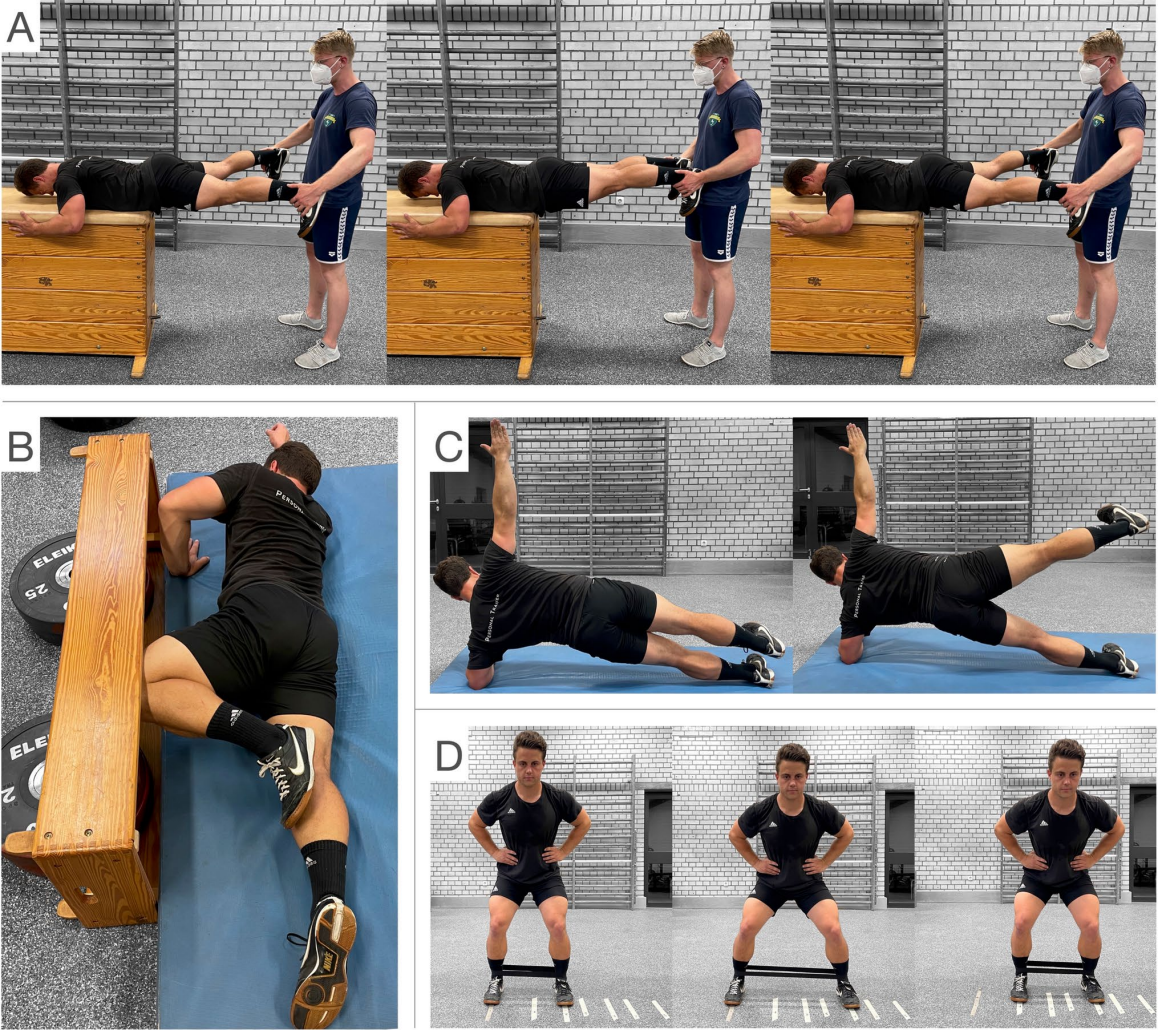
Exemplary setup for the four gluteus medius exercises: **A** Resisted prone hip abduction, starting position (left), position between eccentric and concentric phase (middle) and isometric hold and end position (right); **B** isometric clam with inclined pelvic position; **C** side-plank with leg abduction, adducted (left) and abducted position (right); **D** resisted side-stepping, starting position (left), position after abduction (middle) and reproduced starting position (right)

A cross-sectional within-subjects design was used to compare muscle activity of the gluteal muscles between two traditional strength training and two acceleration-specific exercises for both, GMAX and GMED. For GMAX, the hip thrust and split squat were performed as traditional exercises and compared with the acceleration-specific exercises half-kneeling glute squeeze and resisted knee split (Fig. 1, Table 1). The traditional GMED exercises side-plank with leg abduction and resisted side-stepping were compared with the acceleration-specific resisted prone hip abduction and isometric clam (Fig. 2, Table 1). Detailed information about the standardized criteria is provided in the supplementary materials (A.2). All participants completed two sessions separated by at least 5 and a maximum of 10 days.

**Table 1:** Exercise characteristics and additional information of the four gluteus maximus (top) and the four gluteus medius (bottom) exercises

Exercise	Contraction mode	Tut [s]	Repetitions	Supplementary information
RKS	iso	3	1	Maximum voluntary contraction of dominant GMAX
				Maximum isometric hip flexion of non-dominant limb
HKGS	iso-iso-iso	3-3-3	1	Maximum voluntary contraction of dominant GMAX during all three phases
HT	con-iso-ecc	1-1-1	3	3-RM
SS	ecc-con	1-1	3	3-RM
RPHA	ecc-con-iso	3-2-1	1	Maximum voluntary abduction during all three phases
IC	iso	3	1	Maximum voluntary isometric external rotation of dominant limb
SP	iso-iso	1-1	3	Abduction of the non-dominant limb
RSS	con-ecc	1-1	3/3	Three steps towards both sides

* 3-RM* three-repetition maximum, *con* concentric, *ecc* eccentric, *GMAX* gluteus maximus, *GMED* gluteus medius, *HKGS* half-kneeling glute squeeze, *HT* hip thrust, *IC* isometric clam, *iso* isometric, *RKS* resisted knee split, *RPHA* resisted prone hip abduction, *RSS* resisted side-stepping, *SP* side-plank with leg abduction, *SS* split squat, *tut* time under tension

The familiarization session started with the collection of anthropometric data (weight, height, leg length, shoulder width) and a standardized warm-up. The warm-up included 5 min of self-paced low-intensity jogging and a dynamic movement preparation focusing the lower body. The participant’s three-repetition maximum (3-RM) was tested for the hip thrust and the split squat in randomized order according to the National Strength and Conditioning Association (Haff and Triplett 2016). Furthermore, the participants were familiarized with the other six exercises in detail until the examiner confirmed the quality of execution.

In the experimental session, the same warm-up was applied before the electrodes were placed and a quality check of the signal was conducted. Subsequently, maximum voluntary isometric contraction (MVIC) trials were performed. According to the results of block randomization, participants started with either the GMAX or GMED block. Each block started with the MVIC trials of the respective muscle followed by the four exercises in a randomized order. After the first block, the MVIC testing and the four exercises in a randomized order of the second muscle were performed. To reduce intra-session fatigue and promote maximum possible activity levels, the participants performed each exercise only once as long the form was accurate, and the EMG signal was recorded correctly. Between exercises, four minutes of rest were provided. Prior to the hip thrust and the split squat, a set of three repetitions was conducted with 60% of the 3-RM load. For all exercises, the EMG activity of the dominant limb was recorded. A speaker provided a standardized timer to ensure accurate time under tension (Table 1).

### Electromyography

#### Data recording

Wireless surface EMG electrodes (Delsys^®^ TrignoTM, Boston, MA) were used to record muscle activity. Skin preparation and electrode placement were conducted according to the guidelines of the project for surface electromyography for the non-invasive assessment of muscles (SENIAM) on the dominant limb (Hermens et al. 2000). The skin was shaved and slightly roughened with a hand razor and sterilized with alcohol before the electrodes were fixed using double-sided tape and Fixomull stretch (BSN medical, Hamburg, Germany).

For GMAX MVIC testing, participants lay in the prone position and extended their hip against manual resistance applied to the distal end of the thigh with 90° of knee flexion (Hermens et al. 2000; Worrell et al. 2001). GMED MVIC was determined in a side-lying position with the lower hip and knee joint in 30° of flexion for increased stability. The upper leg was elevated to approximately 10° before performing maximum abduction against manual resistance applied to the distal end of the thigh and the shank (Hermens et al. 2000; Widler et al. 2009). For both muscles, three attempts of 5 s were conducted under verbal encouragement of the examiner with 60 s of recovery after each trial (Boren et al. 2011).

#### Data processing

EMGworks^®^ Acquisition (Delsys, Natick, MA, USA) was used to record the 2000 Hz electromyographic signal, which was exported as raw data and imported to R-Studio (Version 1.4.1106, PBC, Boston, USA) for further processing. The surface EMG raw data was rectified, band pass filtered between 20 and 500 Hz using a Butterworth 4th order recursive filter (Eliassen et al. 2018) and smoothed using root-mean-square with a 100 ms window. Peak data of the exercises were normalized to a mean of a 1000 ms window from the peak value of the three MVIC trials (Contreras et al. 2015).

### Statistical analysis

All calculations were performed using R-Studio. EMG data are presented as mean and 95% confidence interval (CI) of the peak %MVICs. Statistical significance was set at *p* ≤ 0.05. For all data, normal distribution was assessed by the Shapiro–Wilk test and sphericity of data was tested using Mauchly’s test. A one-way repeated-measures analysis of variance (ANOVA) was used to analyze the differences between exercises. Partial eta squared (*η*_*p*_^*p2*^) was calculated with 95% CI as effect size and interpreted upon the guidelines of Cohen with *η*_*p*_^*2*^ ≥ 0.26 large; 0.26–0.13 moderate; 0.13–0.02 small; < 0.02 negligible (Cohen 2013). Bonferroni post hoc tests were used for pairwise comparisons between exercises. Cohen’s d effect sizes are reported with 95% CI for all pairwise comparisons interpreted as *d* ≥ 0.8 large; 0.8–0.5 moderate; 0.5–0.2 small; < 0.2 negligible (Cohen 2013).

## Results

**Fig. 3:**
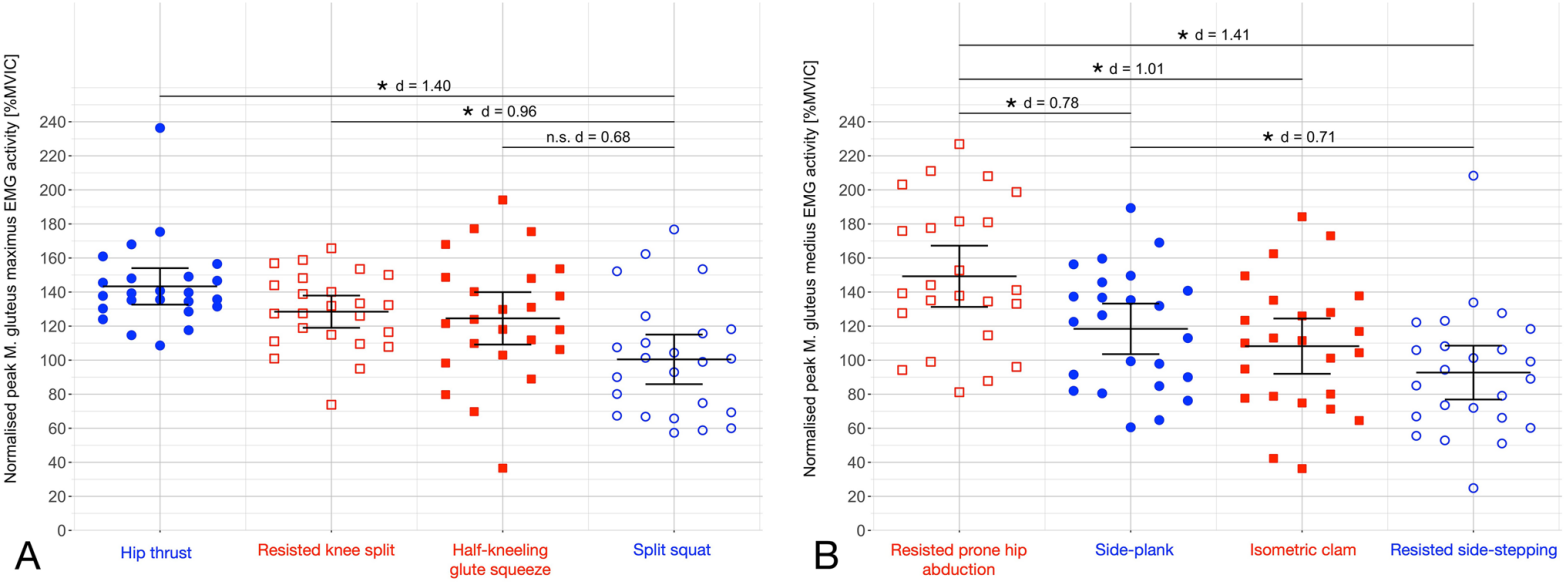
Individual peak electromyographic activities expressed as a percentage of maximum voluntary isometric contraction with mean and 95% confidence interval for all gluteus maximus (**A**) and gluteus medius (**B**) exercises. The acceleration-specific exercises are emphasized in red, the traditional strength training exercises in blue. *Significant difference with *p* ≤ 0.05; *d* = Cohen’s d effect size

**Fig. 4:**
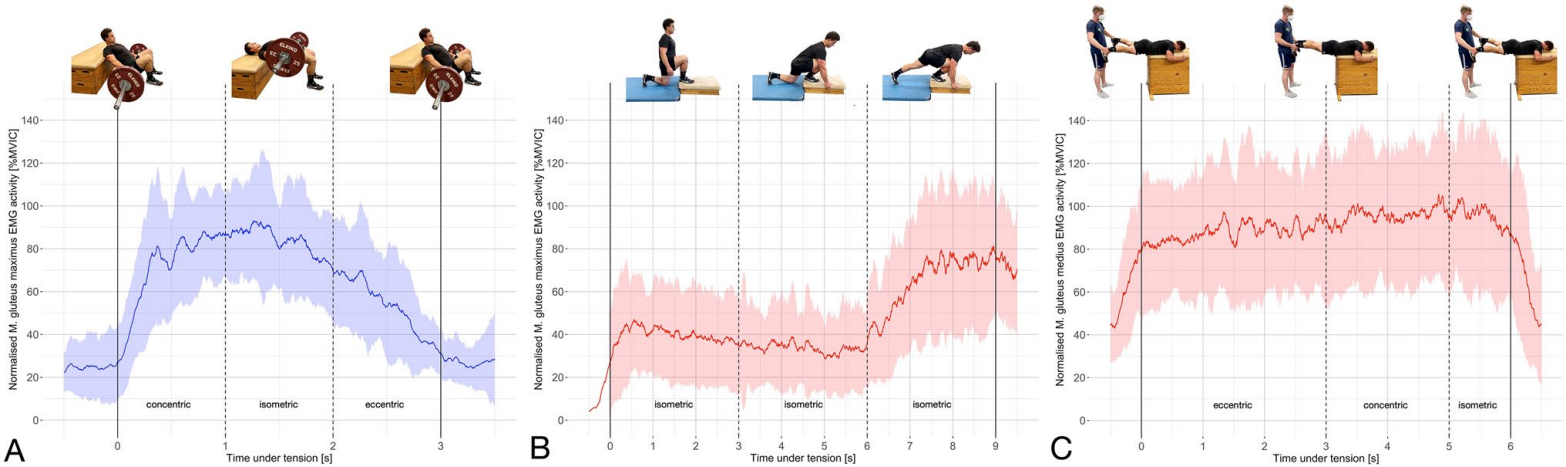
Mean electromyographic activity ± standard deviation expressed as a percentage of maximum voluntary isometric contraction for the different phases of the hip thrust (**A**), half-kneeling glute squeeze (**B**) and resisted prone hip abduction (**C**)

For the two traditional strength training and the two acceleration-specific exercises of each muscle, the EMG data of all *n* = 24 participants were analyzed and included. The mean load for the 3-RM hip thrust (154 ± 51 kg) was twice as high as for the split squat (77 ± 28 kg, *p* < 0.001). EMG data are displayed as peak activity with 95% CI averaged across all participants (Table 2) and individual peak activities expressed as %MVIC (Table 3, Fig. 3). The time courses of the hip thrust, resisted prone hip abduction and half-kneeling glute squeeze are presented as mean ± standard deviation to identify the different phases of the exercises (Fig. 4).

**Table 2:** Mean and 95% CI of peak EMG activity expressed as a percentage of maximum voluntary isometric contraction for the gluteus maximus and medius exercises with *p*-values and Cohen’s d effect sizes of the pairwise comparisons

**Muscle**	**Exercise**	**Mean ** [%MVIC]	**95% CI** [%MVIC]	**Pairwise comparisons**			
				HT (3-RM)	RKS	HKGS	SS (3-RM)
GMAX	HT (3-RM)	143	133–154		*p* = 0.118; d = 0.62	*p* = 0.135; d = 0.59	*p* < 0.001*; d = 1.40
	RKS	128	119–138	*p* = 0.118; d = 0.62		*p* = 1.000; d = 0.12	*p* = 0.011*; d = 0.96
	HKGS	125	109–140	*p* = 0.135; *d* = 0.59	*p* = 1.000; *d* = 0.12		*p* = 0.064; *d* = 0.68
	SS (3-RM)	100	86–115	*p* < 0.001*; *d* = 1.40	*p* = 0.011*; *d* = 0.96	*p* = 0.064; *d* = 0.68	
GMED				RPHA	SP	IC	RSS
	RPHA	149	131–167		*p* = 0.002*; *d* = 0.78	*p* = 0.001*; *d* = 1.01	*p* < 0.001*; *d* = 1.41
	SP	118	104–133	*p* = 0.002*; *d* = 0.78		*p* = 1.000; *d* = 0.28	*p* = 0.040*; *d* = 0.71
	IC	108	92 - 124	*p* = 0.001*; *d* = 1.01	*p* = 1.000; d = 0.28		*p* = 0.338; *d* = 0.41
	RSS	93	77–109	*p* < 0.001*; *d* = 1.41	*p* = 0.040*; *d* = 0.71	*p* = 0.338; *d* = 0.41	

Significant differences (*p* ≤ 0.05) between exercises are emphasized *

The 95% CIs for Cohen’s d effect sizes are provided in the supplementary data (A.3.2)

*GMAX Gluteus maximus, GMED gluteus medius MVIC* maximum voluntary isometric contraction, *CI* confidence interval, *HT* hip thrust, *RKS* resisted knee split, *HKGS* half-kneeling glute squeeze, *SS* split squat, *RPHA* resisted prone hip abduction, *SP* side-plank, *IC* isometric clam, *RSS* resisted side-stepping

**Table 3:**
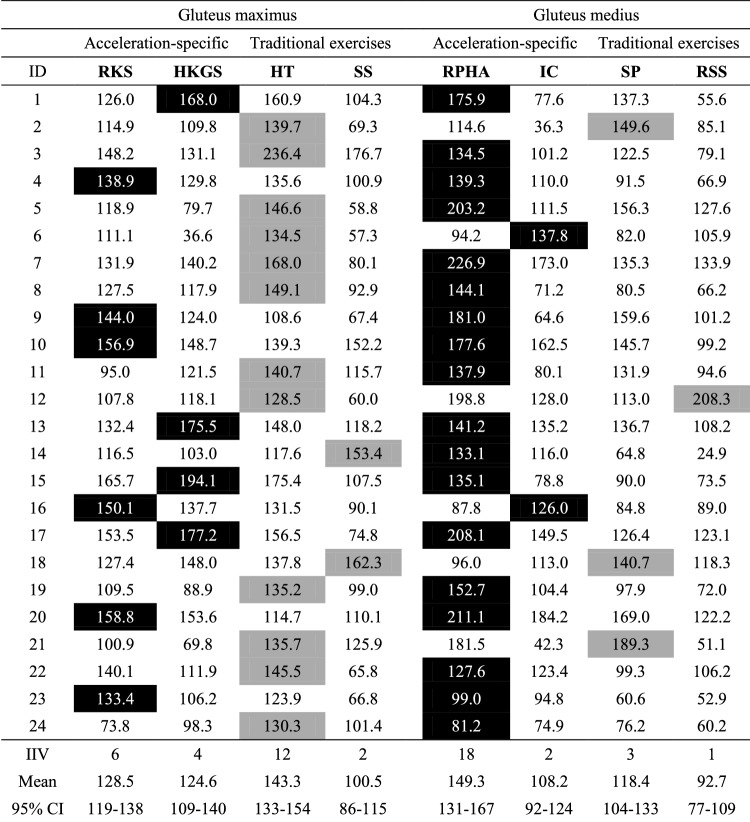
Individual peak EMG activity expressed as a percentage of maximum voluntary isometric contraction for the four gluteus maximus (left) and the four gluteus medius (right) exercises

The peak activity of each participant for each muscle is emphasized (black for the acceleration-specific, gray for the traditional exercises) and totaled as intra-individual variability

CI confidence interval, HKGS half-kneeling glute squeeze, HT hip thrust, IC isometric clam, IIV Intra-individual variability, RKS resisted knee split, RPHA resisted prone hip abduction, RSS resisted side-stepping, SP side-plank with leg abduction, SS split squat

### Gluteus maximus

For the GMAX, one-way repeated-measures ANOVA revealed a significant main effect for the factor exercise with a large effect size (*η*_*p*_^*2*^ = 0.34, 95% CI = 0.16–0.48; *p* < 0.001). No significant differences were found between the hip thrust, resisted knee split and half-kneeling glute squeeze. The hip thrust (*d* = 1.40, 95% CI = 0.74–2.06; *p* < 0.001) and the resisted knee split (*d* = 0.96, 95% CI = 0.30–1.62; *p* = 0.011) resulted in significantly higher peak GMAX activity than the split squat with large effect sizes. The half-kneeling glute squeeze failed statistical significance but showed a moderate effect size compared to the split squat (*d* = 0.68, 95% CI = 0.13–1.22; *p* = 0.064) (Table 2, Fig. 3A). The intra-individual analysis for the GMAX exercises demonstrated that 42% of the participants achieved the highest activity in one of the acceleration-specific exercises. Twelve participants elicited peak activity in the hip thrust, six participants in the resisted knee split, four participants in the half-kneeling glute squeeze and two participants in the split squat (Table 3).

### Gluteus medius

Concerning the GMED, one-way repeated-measures ANOVA revealed a significant main effect for the factor exercise with a large effect size (*η*_*p*_^*2*^ = 0.39, 95% CI = 0.20–0.53; *p* < 0.001). The resisted prone hip abduction demonstrated significantly higher peak activity than resisted side-stepping (*d* = 1.41, 95% CI = 0.80–2.01; *p* < 0.001), the side-plank with leg abduction (*d* = 0.78, 95% CI = 0.36–1.20, *p* = 0.0016) and the isometric clam (*d* = 1.01, 95% CI = 0.44–1.58; *p* = 0.0014). The side-plank revealed significantly higher peak activity than resisted side-stepping (*d* = 0.71, 95% CI = 0.17–1.24; *p* = 0.0395) (Table 2, Fig. 3B). The highest activity of the GMED was generated in 83% of the participants during one of the acceleration-specific exercises (18 resisted prone hip abduction, 3 side-plank with leg abduction, 2 isometric clam, 1 resisted side-stepping) (Table 3).

## Discussion

The current investigation examined the EMG activity of four acceleration-specific exercises for the GMAX and GMED. For each muscle, two traditional strength training exercises were compared to two acceleration-specific exercises. The hypothesis was that peak EMG activity is higher during the acceleration-specific compared to the traditional strength training exercises. The highest peak GMAX activity averaged over all participants was found for the hip thrust (143% MVIC) with no significant difference to the resisted knee split (128% MVIC) and half-kneeling glute squeeze (125% MVIC). The hip thrust and the resisted knee split elicited significantly higher peak EMG activity than the split squat (100% MVIC) with large effect sizes (Table 2, Fig. 3A). Therefore, the hypothesis was not confirmed for GMAX. However, 42% of all participants achieved the highest peak GMAX activity during one of the acceleration-specific exercises (Table 3). For GMED, the hypothesis was approved because the resisted prone hip abduction elicited the highest peak EMG activity (149% MVIC) with a significant difference and large effect compared to resisted side-stepping (93% MVIC) and moderate effect compared to the side-plank with leg abduction (118% MVIC) (Table 2, Fig. 3B).

### Acceleration-specific gluteus maximus activation

The high GMAX activity during the acceleration-specific exercises is very promising, especially in terms of the high coordinative transfer to horizontal acceleration, which is recommended for pre-activation and strength training tasks (Smith et al. 2014; Walker et al. 2021). The resisted knee split is performed solely isometric and supports the acceleration-specific position by adding isometric contraction of the contralateral hip flexor muscles during maximum activation of the GMAX (Fig. 1B). The half-kneeling glute squeeze consists of three phases with an increasing contralateral hip flexion during the second phase. This dynamic movement makes it more difficult to achieve a sustained high level of GMAX activity which explains the decreasing activity in phase two. Mean peak activity occurs in the third phase when the dominant knee is extended (Fig. 4B).

This final position of the half-kneeling glute squeeze induces a large thigh separation and a low trunk angle, which have been demonstrated as vital components of enhanced acceleration performance (Walker et al. 2021). The thigh separation angle depends on the range of motion of hip extension in the stance leg and concomitant hip flexion in the swing leg (Alt et al. 2022). Co-contraction of the antagonistic muscles during activation of the agonistic muscles is a common phenomenon (Frey-Law and Avin 2013). The theory of muscular stabilization is a potential mechanism responsible for the increase in hip flexor co-contraction during GMAX activation with increasing separation of the thighs (Thorborg et al. 2016). High iliacus activity in the late stance phase (Andersson et al. 1997) and high psoas major activity during contralateral hip flexion (Hu et al. 2010) have been found in past investigations. Reciprocal inhibition results in decreased GMAX activity in the stance leg and impairs hip flexion in the swing leg (Mills et al. 2015). The decreasing GMAX activity during increasing contralateral hip flexion in the second phase of the half-kneeling glute squeeze emphasized this distinctive activation pattern (Fig. 4B). Both, lower GMAX activity and decreased hip flexion range of motion result in a smaller thigh separation angle and might impair performance. Reduced hip flexor tightness enhanced GMAX activity, pelvic position and performance (Konrad et al. 2021).

The acceleration-specific exercises focus on ’reciprocal activation’ of the GMAX which is intended to enhance the following characteristics:

1. Ipsilateral hip extension: Higher levels of GMAX activity enable improved control of ipsilateral hip extension through reduced co-contraction of the ipsilateral hip flexor muscles (Mills et al. 2015; Neumann 2010).
2. Contralateral hip flexion: The decrease in hip flexor co-contraction is suggested to enable an increase in contralateral hip flexion range of motion due to improved muscular stabilization of the pelvis (Thorborg et al. 2016). This might further support a decrease in activity of the adductor magnus, which functions as a hip extensor, especially when the hip is in a flexed position (Ward et al. 2010). The adductor magnus has been shown to remain strongly excitated during the swing phase which might inhibit hip flexion range of motion (Gazendam and Hof 2007).
3. Anterior pelvic tilt: The sustained high level of GMAX activity is suggested to enhance dynamic control of the anterior pelvic tilt (Neumann 2010), which is a common consequence of insufficient hip extension capability during maximal sprinting (Schache et al. 2000). High pelvic control through a synergistic interaction of concomitant hip flexion and extension might improve the trunk position during sprinting, which results in a more horizontally orientated force vector (Walker et al. 2021).

This simultaneous enhancement of ipsilateral hip extension and contralateral hip flexion ability could improve both the thigh separation angle and ‘scissor-action’ of the thighs due to increased thigh angular velocity (Clark et al. 2020).

Furthermore, reciprocal inhibition of the GMAX has been shown to increase the risk of injury to the hamstring and adductor muscles and anterior cruciate ligament through synergistic dominance. Increasing the GMAX activity may help reduce the reliance on the secondary hip extensors and improve the balance between the hamstring, hip adductor and gluteal muscles in generating hip extension torque (Mills et al. 2015). In addition, a decrease in hip flexor co-contraction is thought to reduce the injury prevalence of the hip flexors. Overuse injuries are very common during sports that require maximal sprinting and are exacerbated by reciprocal inhibition of the GMAX (Thorborg et al. 2016).

The previously mentioned acceleration-specific activation patterns are not mirrored in traditional strength training exercises. However, the hip thrust and the split squat elicited high GMAX activity, which is in line with past findings (Contreras et al. 2015; Neto et al. 2020; Williams et al. 2021). The horizontal force-vector direction and the peak activity occurring during the isometric phase with a neutral hip position (Fig. 4A) support the use of the hip thrust in strength training programs to improve horizontal acceleration (Loturco et al. 2018; Williams et al. 2021). The free barbell split squat demands a high level of core and lower limb stability which might have caused lower GMAX activity in some participants (Macadam and Feser 2019).

### Higher peak gluteus medius activity during acceleration-specific exercises

Of all participants, 83% achieved the highest peak activity in the GMED during one of the acceleration-specific exercises (Table 3), which supports their use to elicit high-intensity muscle contractions. These high intensities of the GMED are important for horizontal acceleration performance for the following threereasons:

1. Hip stabilization: The GMED is the most important hip stabilizer in the frontal plane (Neumann 2010). A lack of hip stabilization during the stance phase causes a lateral tilt of the pelvis, causing a shift of the body’s center of mass that impairs force transmission of the lower limb and increases the risk of injuries (Presswood et al. 2008).
2. Activation ratio between abductors and adductors: Groin injuries among athletes caused by overuse are very common (Eckard et al. 2017). Higher GMED activity in the late swing phase was associated with lower peak adduction angles in the early stance phase (Chumanov et al. 2012). It is suggested that high GMED activity during sprinting improves the adductor-abductor ratio and lowers the risk of overuse injuries of the adductor muscles. Furthermore, this might help increase the thigh separation angle due to improved hip flexion range of motion in the swing leg as described earlier in this paper.
3. Secondary hip extensor: The GMED functions as a secondary hip extensor and thus supports high GMAX activation (Neumann 2010). This has been shown to be essential during accelerated sprinting, as the GMED acts as an important generator of propulsive impulses (Pandy et al. 2021).

The mean activity during the resisted prone hip abduction was found to be high during the entire time under tension, irrespective of muscle length and type of contraction (Fig. 4C). During sprinting, the GMED primarily works isometrically in a position of approximately 10° of adduction (Neumann 2010). Consequently, the exercise can easily be adjusted to highly activate the GMED during an extended and adducted hip position as it is necessary for horizontal acceleration.

The contraction of the GMED during the isometric clam exercise is primarily induced by external rotation. This supports a higher GMED activity compared to the tensor fasciae latae (Selkowitz et al. 2013). A higher inclination of the pelvis improves GMED activity during the clam exercise (Willcox and Burden 2013). Therefore, the isometric clam in this investigation was performed with a high inclination of the pelvis (Fig. 2B). However, performing maximum isometric external rotation without reclining the pelvis was challenging for some participants, which may have limited higher activity levels. The peak GMED activity of the isometric clam might increase when athletes are familiar with the exercise.

The high muscle activity during the side plank with leg abduction confirms the results of past investigations (Boren et al. 2011). The isometric GMED contraction in the lower leg is intensified by the upward acceleration of the non-dominant leg during abduction when compared to normal side planks (Ekstrom et al. 2007). The high muscular activity and the stabilizing role in a neutral adducted and extended hip position make the exercise interesting for sprint training approaches (Hamner et al. 2010). With the modification of abduction speed and external loads, the side plank with leg abduction might be customized to suit different training concepts of the GMED. The resisted side-stepping elicited the lowest peak GMED activity (93% MVIC), although it was performed with the best possible execution criteria concerning side-step length and resistance. Therefore, it seems reasonable to suggest that it is hardly possible to achieve higher muscular activities during this exercise than in the present investigation. The resisted side-stepping might be an appropriate exercise in rehabilitation settings, but not to highly activate the GMED in athletes.

### Enhanced acceleration performance after gluteal pre-activation

The investigation confirmed that the acceleration-specific exercises elicited very high muscle activity for both GMAX and GMED. Accordingly, they might be suited for high-intensity pre-activation in training sessions and competitions. Especially in sports that require the generation of maximum power, such as multiple track and field disciplines, but also in team sports such as soccer, American football, basketball or ice hockey, the use of PAP in competitions could be a crucial factor for performance (DeRenne 2010). A high association between hip thrust pre-activation and the acceleration phase (up to 10 m) supports the effect of GMAX PAP on horizontal acceleration (Loturco et al. 2018; Neto et al. 2019). Dello Iacono et al. (2018) described the importance of sport-specific pre-activation tasks and supported the use of hip thrusts before sprinting due to their horizontal force vectors. The acceleration-specific exercises for the GMAX are highly specific and might therefore be even more beneficial as pre-activation tasks. Furthermore, as demonstrated in this study, very high external loads are required to elicit the highest amount of muscle activity with the hip thrust or similar strength training exercises (mean 3-RM load: 154 ± 51 kg) (Dello Iacono et al. 2018). This is usually possible in separate strength training sessions, but prior to sprinting sessions or even competitions, the high amount of equipment might be a logistical challenge. The introduced acceleration-specific exercises require little equipment and setup and might therefore be preferential for pre-activation tasks. Furthermore, a high number of athletes can simultaneously perform the exercises, which is of great advantage for team sports. The acceleration-specific exercises can complement the warm-up process by increasing activation levels to maximum pre-activation and thus provide an appropriate balance between potentiation and accumulating fatigue (Rassier and Macintosh 2000; Seitz and Haff 2016).

### The acceleration-specific exercises in daily training routines

Besides the use as pre-activation, the acceleration-specific exercises are also intended to be used in daily training routines to strengthen the gluteal muscles and improve task-specific activation patterns. Isometric training has been shown to elicit neurological and morphological adaptations (Oranchuk et al. 2019), which also transfer to dynamic movement tasks (Burgess et al. 2007). Significant improvements in GMAX activity during double-leg and single-leg squats after an isometric activation program have been demonstrated (Cannon et al. 2022). The acceleration-specific exercises are not intended to replace traditional strength training exercises with external loads, but to act mutually supportive. They reveal new opportunities to improve acceleration-specific activation, maximum strength of GMAX and GMED and lower extremity injury prevention. Due to the high muscle activity, they might also support tendon adaptations and increase muscular stiffness to improve the transmission of force between muscle and bone (Brazier et al. 2019; Oranchuk et al. 2019).

The findings of this study are also of interest for rehabilitation. Especially in the early stages of rehabilitation, high-intensity isometric contractions have been suggested to improve and maintain motor unit recruitment. Due to higher voluntary muscle activations compared to concentric and eccentric contractions, isometric training could help prevent post-injury activation deficits (Macdonald et al. 2019). The acceleration-specific exercises enable voluntary activation at various activity levels up to maximum intensities with very low joint stress. Therefore, they might help maintain gluteal strength and neuromuscular control after hip joint or hamstring injuries.

### Limitations and perspectives

The results of this study rely on a very heterogeneous group of participants from various athletic backgrounds with different performance levels and strength training experience (supplementary data A.1). The participants had only one familiarization session and were not used to the acceleration-specific or similar exercises. In addition, only one set of each exercise was performed during the experimental session due to the risk of accumulating fatigue. Especially the half-kneeling glute squeeze and the isometric clam revealed a high inter-individual variability which is suggested to be caused by the high coordinative demands (Table 3). A more homogeneous sample with high-performance athletes who are experienced in these exercises could specify the results.

This investigation did not determine hip flexor muscle activity, because it is hardly possible with surface EMG (Andersson et al. 1997; Hu et al. 2010). However, the extent of hip flexor co-contraction in the maximum possible thigh separation angle and its alteration through acceleration-specific exercises should be investigated. Furthermore, the transfer of the exercises to horizontal acceleration is not yet known. An intervention study is needed to examine the effects of the acceleration-specific exercises on GMAX and intramuscular hip flexor EMG activity during acceleration as well as maximum velocity sprints, and its influence on performance. In addition, potential acceleration performance enhancements after pre-activation with these exercises and the influence on overuse injury prevalence of the hip flexor, hamstring and adductor muscles should be part of further investigations. In case of additional evidence of the acceleration-specific exercises, transferring this training approach to other muscle groups like the hamstrings, calves or shoulders seems feasible.

Seven out of eight exercises determined in this investigation achieved values greater than 100% of MVIC. Although similar results for peak EMG values were found in past investigations (Boren et al. 2011; Contreras et al. 2015; Williams et al. 2021), the MVIC testing positions should be questioned to allow more reliable classifications of exercise intensities. Concerning normalization methods, but also for the acceleration-specific exercises, visual feedback should be considered. Providing visual feedback in the form of a real-time EMG activity graph while voluntary activations are performed has been shown to increase activity levels (Amagliani et al. 2010). In addition to visual feedback, surface EMG could also be used as a training tool to ascertain which exercises work best for each individual and where the greatest potentials are in terms of acceleration-specific activation patterns. Further research with a more detailed analysis of inter-individual variabilities is needed to determine adaptations after exercises that elicit different EMG activities.

## Conclusion

Despite the lack of participants’ experience in the acceleration-specific exercises, 42% and 83% achieved the highest peak activity of the GMAX and GMED, respectively, during one of these exercises. Ipsilateral hip extension and concomitant contralateral hip flexion decisively determine the thigh separation angle, which is essential for horizontal acceleration performance. In contrast to traditional strength training, the acceleration-specific exercises mirror this specific activation pattern of the gluteal muscles, which is suggested to transfer to horizontal acceleration. Given the high activity levels with low joint stress and minimal equipment requirements, the exercises appear to be suitable for task-specific pre-activation, strengthening and rehabilitation of the gluteal muscles. In addition, they might support the prevention of lower extremity injuries due to improved activation ratios of the hip muscles. After the acceleration-specific exercises have been practically approved, this study provides the first scientific findings. Further intervention studies are needed to examine the direct transfer to acceleration performance after training and pre-activation with the acceleration-specific exercises.

### Supplementary Information

Below is the link to the electronic supplementary material.

Supplementary file1 (PDF 178 kb)

## Data Availability

A data availability statement is not applicable, however data is provided in the supplementary information.

## Abbreviations

3-RM: Three-repetition maximum
ANOVA: Analysis of variance
CI: Confidence interval
EMG: Electromyography
GMAX: Gluteus maximus
GMED: Gluteus medius
MVIC: Maximum voluntary isometric contraction
PAP: Post-activation potentiation
SENIAM: Surface electromyography for the non-invasive assessment of muscles
