# Supplementary material for: How to activate the glutes best? Peak muscle activity of acceleration-specific pre-activation and traditional strength training exercises
Source: Eur J Appl Physiol. 2024 Jan 27;124(6):1757–69. doi: 10.1007/s00421-023-05400-3 (PMC11130056; doi:10.1007/s00421-023-05400-3)
Supplement: Supplementary file 1 — Supplementary file1 (PDF 178 kb) [file 421_2023_5400_MOESM1_ESM.pdf]

Supplementary data

How to activate the glutes best?
Peak muscle activity of acceleration-specific pre-activation and traditional strength training exercises

European Journal of Applied Physiology

Maximilian Goller[a*], Oliver J. Quittmann[a], Tobias Alt[b]

[a] Institute of Movement and Neurosciences, German Sport University Cologne, Cologne, Germany
[b] Department of Biomechanics, Performance Analysis and Strength & Conditioning, Olympic Training and Testing Centre Westphalia, Dortmund, Germany

*corresponding author:
Maximilian Goller
Rhöndorfer Straße 8, 50939 Köln
E-mail: goller.sportsscience@web.de

A Supplementary data

A.1 Supplementary participant information

Table A.1: Sex, weight, height, strength training experience, 3-RM hip thrust load, 3-RM split squat load, leg length, shoulder width, mini-band colour for the resisted side-stepping, dominant leg and athletic background of all participants.

ID	Sex	Age (years)	Weight (kg)	Height (cm)	STE (years)	3-RM HT (kg)	3-RM SS (kg)	Leg length (cm)	Shoulder width (cm)	Mini-band colour	Dominant leg	Athletic background
1	m	24	80.8	182	3.5	165	80	85	43	red	left	soccer
2	f	25	66.5	174	8.0	110	60	80	38	black	left	soccer
3	m	25	87.3	168	8.0	220	125	76	47	black	left	soccer
4	f	29	68.1	167	3.0	120	55	80	36	black	right	handball
5	f	24	62.5	173	1.5	110	50	84	42	red	right	handball
6	m	25	87.8	190	6.0	220	90	91	43	black	right	soccer
7	m	32	78.9	180	5.0	150	75	88	47	black	left	calisthenics
8	f	22	57.4	169	1.0	100	50	87	37	red	right	indiaca
9	m	23	85.5	187	6.0	160	90	91	46	black	right	basketball
10	m	24	88.8	188	8.0	220	115	84	42	black	left	handball
11	m	28	98.2	198	7.0	145	70	94	44	black	left	boxing
12	m	28	76.3	173	10.0	160	105	76	41	black	right	Olympic weightlifting
13	f	24	61.7	170	3.0	100	40	86	37	red	right	pole vault
14	m	24	80.9	188	1.0	160	80	91	44	black	right	beachvolleyball
15	f	21	77.2	180	5.0	145	75	85	44	red	right	gymnastics
16	m	25	82.0	181	8.0	160	100	87	40	black	right	soccer
17	f	35	65.1	165	2.0	110	45	78	39	red	right	yoga
18	m	27	91.1	185	10.0	140	70	90	43	black	right	soccer
19	f	21	64.6	162	2.0	115	55	76	38	red	left	marathon running
20	m	24	102.9	184	5.0	265	120	84	45	black	left	American football
21	m	26	97.3	188	8.0	270	135	83	46	black	left	American football
22	f	27	62.8	174	8.0	110	50	87	39	red	right	badminton / beachvolleyball
23	f	21	56.1	164	3.0	130	50	81	36	black	right	middle-distance running
24	f	30	60.7	176	10.0	100	55	88	39	red	left	pole vault
Mean		25.6	76.7	177.8	5.5	153.5	76.7	84.7	41.5			
SD		3.5	13.9	9.6	3.0	50.7	27.8	5.1	3.5			

Footnote: SD (standard deviation), STE (strength training experience), 3-RM (three-repetition-maximum), HT (hip thrust), SS (split squat)

A.2 Detailed exercise descriptions

A.2.1 Gluteus maximus exercises

The **half-kneeling glute squeeze** consisted of three phases and was performed with the dominant leg behind and the non-dominant leg elevated on a box with 13 cm height. Phase one involved the half-kneeling position with the tibia of the anterior leg and the femur of the posterior leg vertical to the ground. In phase two an 'ankle rocker' to maximum dorsal flexion without tilt of the hip in the transversal plane was performed. At the end of phase two, participants grabbed the box by keeping the dominant hip and the back as extended as possible. In phase three, the shin of the non-dominant leg was tilted downwards ('shin roll') and the knee of the dominant leg was extended with the heel pushing backwards and upwards (Figure 1A) (Alt et al. 2022). Each of the three phases consisted of 3 seconds following a constant beat of 9 seconds. Participants were encouraged to activate the dominant gluteus maximus as strong as possible throughout the entire time under tension.

The **resisted knee split** was performed in a standing position with 0° hip angle in the dominant leg and isometric hip flexion of the non-dominant leg (Figure 1B). The height of the box was 106 ± 6 cm and the distance between the toes of the dominant leg and the box was 67 ± 6 cm. The height and the distance were adjusted individually according to the participant's perception to reach a high activation of the gluteus maximus. The heel of the posterior leg had to maintain contact with the ground and the knee had to remain fully extended. The knee angle of the non-dominat leg was ≤ 90°. Participants were encouraged to activate the dominant gluteus maximus as strong as possible and to push the non-dominant leg as strong as possible upwards against the resistance (isometric hip flexion). Three seconds of increasing pre-activation was followed by 3 seconds of maximum activation to a beat of 6 seconds.

The **split squat** was conducted with the barbell above the trapezius muscle in a high-bar position and with the dominant leg in front. The individual step length was obtained by using 85% of the participant's leg length (Schütz et al. 2014). Participants were instructed to align their anterior knee perpendicular to their toes in the end of the eccentric phase to achieve a tibia angle of 60-70° (Schütz et al. 2014). The posterior knee had to touch the ground during each repetition (Figure 1C). Three repetitions with the 3-RM load were performed according to a beat counting of one-two for the concentric and eccentric phases, respectively.

The **hip thrust** was conducted with a padded barbell according to (Contreras et al. 2011). The feet were placed shoulder width apart, creating a 90° knee angle with the shin vertical to the ground when the hip is extended. The upper back was placed on a box with a height of 45cm. The barbell was raised until the torso was parallel to the ground (Figure 1D). Three repetitions with the 3-RM were performed according to a beat counting of one-two-three for each repetition. Participants were instructed to perform one second of concentric, one second of isometric and one second of eccentric contractions for each repetition.

A.2.2 Gluteus medius exercises

The **resisted prone hip abduction** was performed on a box with the legs distal to the iliac crest in midair. Full hip and knee extension as well as a dorsal ankle flexion was maintained throughout the entire exercise. Starting and end position of the exercise were in maximum abduction. Participants were encouraged to abduct as forcefully as possible against an external resistance for 6 seconds. During the first 3 seconds, the participants legs were pushed into adduction by the examiner (eccentric phase). The following 2 seconds, the legs were abducted against the resistance to reproduce the starting position (concentric phase) in which one second of maximum isometric contraction was performed (Figure 2A).

The **isometric clam** was performed in side-lying position with the dominant hip on top, with approximately 60° of flexion and tilted towards the ground (inclined pelvis position). The heel of the dominant leg was in contact with the non-dominant leg throughout the entire exercise. The hip of the dominant side was externally rotated without tilt of the pelvis away from the ground until contact between the knee and the box has been made (Figure 2B). After 3 seconds of increasing pre-activation, participants were encouraged to push as strongly as possible against the immobile resistance for 3 seconds without moving.

The **side-plank with leg abduction** was conducted with the dominant leg below according to the results of Boren et al. (2011) and Ekstrom et al. (2007). The participants started in a side-plank position while aligning shoulders, hips, knees and ankles. Throughout the entire exercise, only the forearm and the foot of the dominant leg were in contact with the ground. The participants abducted the extended upper leg without lowering the dominant hip for one beat and adducted the leg on the second beat without touching the dominant foot (Figure 2C). Three abductions were performed.

Resisted side-stepping was conducted with a resistance band around the ankles (Lewis et al. 2018). Knees and hips were maintained in 30° of flexion, toes pointed straight forward and the hands were positioned on the hips throughout the entire exercise. Participants started the exercise with their feet shoulder-width apart. On the first beat, the dominant leg was abducted to 160% of shoulder width in a controlled movement (Youdas et al. 2013). On the second beat, the non-dominant leg was adducted to reproduce the shoulder-width position. Three steps were taken to the dominant side and three steps back to the starting position. Floor markings were used to control the individual side-step length (Figure 2D). The strength of the resistance band was defined as the strongest band with which the participants were able to perform a controlled side-step at 200% of shoulder-width.

A.3 Supplementary statistics

Table A.3.1: Results of Mauchly's test for sphericity and the Shapiro-Wilk normality test.

	Mauchly's test for sphericity	Shapiro-Wilk normality test			
**Muscle**		**Exercise**			
Gluteus maximus		HT (3-RM)	RKS	HKGS	SS (3-RM)
	W = 0.70; p = 0.17	W = 0.81; p = 0.00	W = 0.98; p = 0.89	W = 0.99; p = 0.99	W = 0.92; p = 0.07
Gluteus medius		RPHA	SP	IC	RSS
	W = 0.72; p = 0.21	W = 0.95; p = 0.31	W = 0.97; p = 0.55	W = 0.98; p = 0.96	W = 0.93; p = 0.11

Footnote: HT (hip thrust), RKS (resisted knee split), HKGS (half-kneeling glute squeeze), SS (split squat), RAPP (resisted prone hip abduction), SP (side-plank), IC (isometric clam), RSS (resisted side-stepping)

Table A.3.2: Cohen's d effect sizes with 95% confidence interval of pairwise comparisons for the M. gluteus maximus and medius exercises.

**Muscle**	**Exercise**	**Cohen's d effect sizes with 95% CI**			
Gluteus maximus		HT (3-RM)	RKS	HKGS	SS (3-RM)
	HT (3-RM)		d = 0.62 [0.08; 1.17]	d = 0.59 [0.06; 1.12]	d = 1.40 [0.74; 2.06]
	RKS	d = 0.62 [0.08; 1.17]		d = 0.12 [-0.23; 0.47]	d = 0.96 [0.30; 1.62]
	HKGS	d = 0.59 [0.06; 1.12]	d = 0.12 [-0.23; 0.47]		d = 0.68 [0.13; 1.22]
	SS (3-RM)	d = 1.40 [0.74; 2.06]	d = 0.96 [0.30; 1.62]	d = 0.68 [0.13; 1.22]	
Gluteus medius		RPHA	SP	IC	RSS
	RPHA		d = 0.78 [0.36; 1.20]	d = 1.01 [0.44; 1.58]	d = 1.41 [0.80; 2.01]
	SP	d = 0.78 [0.36; 1.20]		d = 0.28 [-0.32; 0.87]	d = 0.71 [0.17; 1.24]
	IC	d = 1.01 [0.44; 1.58]	d = 0.28 [-0.32; 0.87]		d = 0.41 [-0.02; 0.83]
	RSS	d = 1.41 [0.80; 2.01]	d = 0.71 [0.17; 1.24]	d = 0.41 [-0.02; 0.83]	

Footnote: CI (confidence interval), HT (hip thrust), RKS (resisted knee split), HKGS (half-kneeling glute squeeze), SS (split squat), RAPP (resisted prone hip abduction), SP (side-plank), IC (isometric clam), RSS (resisted side-stepping)

References

Alt T, Oeppert TJ, Zedler M, Goldmann J-P, Braunstein B, Willwacher S (2022) A novel guideline for the analysis of linear acceleration mechanics - outlining a conceptual framework of 'shin roll' motion. Sports biomechanics:1–18. doi: 10.1080/14763141.2022.2094827

Boren K, Conrey C, Le Coguic J, Paprocki L, Voight M, Robinson TK (2011) Electromyographic analysis of gluteus medius and gluteus maximus during rehabilitation exercises. International Journal of Sports Physical Therapy 6(3):206–223

Contreras B, Cronin J, Schoenfeld B (2011) Barbell Hip Thrust. Strength & Conditioning Journal 33(5):58–61. doi: 10.1519/SSC.0b013e31822fa09d

Ekstrom RA, Donatelli RA, Carp KC (2007) Electromyographic analysis of core trunk, hip, and thigh muscles during 9 rehabilitation exercises. The Journal of orthopaedic and sports physical therapy 37(12):754–762. doi: 10.2519/jospt.2007.2471

Lewis CL, Foley HD, Lee TS, Berry JW (2018) Hip-Muscle Activity in Men and Women During Resisted Side Stepping With Different Band Positions. Journal of athletic training 53(11):1071–1081. doi: 10.4085/1062-6050-46-16

Schütz P, List R, Zemp R, Schellenberg F, Taylor WR, Lorenzetti S (2014) Joint angles of the ankle, knee, and hip and loading conditions during split squats. Journal of applied biomechanics 30(3):373–380. doi: 10.1123/jab.2013-0175

Youdas JW, Foley BM, Kruger BL, Mangus JM, Tortorelli AM, Madson TJ, Hollman JH (2013) Electromyographic analysis of trunk and hip muscles during resisted lateral band walking. Physiotherapy theory and practice 29(2):113–123. doi: 10.3109/09593985.2012.704492
